# Supplementary material for: Watered-down biodiversity? A comparison of metabarcoding results from DNA extracted from matched water and bulk tissue biomonitoring samples
Source: PLoS One. 2019 Dec 12;14(12):e0225409. doi: 10.1371/journal.pone.0225409 (PMC6907778; doi:10.1371/journal.pone.0225409)
Supplement: S1 Table — Information includes waterbody name, latitude and longitude of sample collection. (DOCX) [file pone.0225409.s001.docx]

**S1 Table. Summary information for the eight sites sampled.** Information includes waterbody name, latitude and longitude of sample collection.

| **Site** | **River** | **Waterbody Name** | **Latitude (N)** | **Longitude (W)** |
| --- | --- | --- | --- | --- |
| PAD1 | Athabasca | Otter Creek | 58.60273 | 111.52612 |
| PAD 3 | Athabasca | Mamawi Bay | 58.56475 | 111.51079 |
| PAD 4 | Athabasca | Mamawi Pond | 58.50773 | 111.51802 |
| PAD 11 | Athabasca | Childs River | 58.6384 | 111.59653 |
| PAD 14 | Peace | Rat Lake | 58.87465 | 111.32484 |
| PAD 33 | Peace | Egg Lake | 58.88236 | 111.39922 |
| PAD 37 | Peace | Rocher River | 58.83234 | 111.28074 |
| PAD 38 | Peace | Horseshoe Slough | 58.86389 | 111.58159 |
