# Supplementary material for: Watered-down biodiversity? A comparison of metabarcoding results from DNA extracted from matched water and bulk tissue biomonitoring samples
Source: PLoS One. 2019 Dec 12;14(12):e0225409. doi: 10.1371/journal.pone.0225409 (PMC6907778; doi:10.1371/journal.pone.0225409)
Supplement: S2 Table — (DOCX) [file pone.0225409.s002.docx]

**Table S1. Summary of reads and ESVs in all taxa**

|  | **AD** |  | **BE** |  |  |
| --- | --- | --- | --- | --- | --- |
|  | **Benthos** | **Water** | **Benthos** | **Water** | **Total** |
| Raw | N/A |  | N/A |  | 48,799,721 x 2 |
| Paired | N/A |  | N/A |  | 42,317,963 |
| Primer Trimmed | 4,377,830 | 345,014 | 4,001,465 | 1,561,360 | 8,724,309 |
| ESVs | 2,581 | 1,099 | 4,831 | 9,571 | 16,841 |
| Reads in ESVs | 2,614,558 | 238,697 | 1,774,386 | 780,079 | 5,407,720 |
| Proportion of raw reads in ESVs (%) | 5.4 | 0.5 | 3.6 | 1.6 | 11.1 |
