# Supplementary material for: Watered-down biodiversity? A comparison of metabarcoding results from DNA extracted from matched water and bulk tissue biomonitoring samples
Source: PLoS One. 2019 Dec 12;14(12):e0225409. doi: 10.1371/journal.pone.0225409 (PMC6907778; doi:10.1371/journal.pone.0225409)
Supplement: S3 Table — (DOCX) [file pone.0225409.s003.docx]

**Table S2. Summary of reads and ESVs assigned to the Arthropoda**

|  | **AD** |  | **BE** |  |  |
| --- | --- | --- | --- | --- | --- |
|  | **Benthos** | **Water** | **Benthos** | **Water** | **Total** |
| ESVs | 1,735 | 280 | 2,398 | 491 | 4,459 |
| Reads in ESVs | 2,541,062 | 174,605 | 1,554,853 | 129,429 | 4,399,949 |
| Proportion of raw reads in ESVs (%) | 5.2 | 0.4 | 3.2 | 0.3 | 9.0 |
| Proportion of all ESVs that are Arthropoda | 67.2 | 25.5 | 49.6 | 5.1 | 26.5 |
| Proportion of all reads in ESVs that are Arthropoda | 97.2 | 73.1 | 87.6 | 16.6 | 81.4 |
