# Supplementary material for: Watered-down biodiversity? A comparison of metabarcoding results from DNA extracted from matched water and bulk tissue biomonitoring samples
Source: PLoS One. 2019 Dec 12;14(12):e0225409. doi: 10.1371/journal.pone.0225409 (PMC6907778; doi:10.1371/journal.pone.0225409)
Supplement: S4 Table — Sample replicates were pooled. No significant beta dispersion was detected within groups (collection method, river). No significant interaction between groups was detected (collection method, river). Summary of PERMANOVA results based on a Sorensen dissimilarity matrix of EPTO ESVs. Significant p-values are in bold. Based on normalized data. (DOCX) [file pone.0225409.s004.docx]

**Table S3. EPTO ESVs can be used to separate rivers using either benthos or water collection methods**

| Source of variation | Df | MS | F | R^2^ | P |
| --- | --- | --- | --- | --- | --- |
| A) Interaction between groups | | | | | |
| Collection method | 1 | 0.80 | 2.09 | 0.13 | **0.002** |
| River | 1 | 0.66 | 1.73 | 0.11 | **0.021** |
| Collection method : River | 1 | 0.44 | 1.16 | 0.07 | 0.282 |
| Residuals | 11 | 0.38 |  | 0.69 |  |
| Total | 14 |  |  | 1.00 |  |
|  |  |  |  |  |  |
| B) Variation due to collection method | | | | | |
| Collection method | 1 | 0.80 | 1.95 | 0.13 | **0.011** |
| Residuals | 13 | 0.41 |  | 0.87 |  |
| Total | 14 |  |  | 1.00 |  |
|  |  |  |  |  |  |
| C) Within each collection method, variation due to river | | | | | |
| River,  Stratum = Collection Method | 1 | 0.65 | 1.56 | 0.11 | **0.031** |
| Residuals | 13 | 0.41 |  |  |  |
| Total | 14 |  |  |  |  |
|  |  |  |  |  |  |

Df = Degrees of freedom; MS = MeanSqs; F = F.Model; P = P-value
