# Supplementary figures and images for: Watered-down biodiversity? A comparison of metabarcoding results from DNA extracted from matched water and bulk tissue biomonitoring samples
Source: PLoS One. 2019 Dec 12;14(12):e0225409. doi: 10.1371/journal.pone.0225409 (PMC6907778; doi:10.1371/journal.pone.0225409)

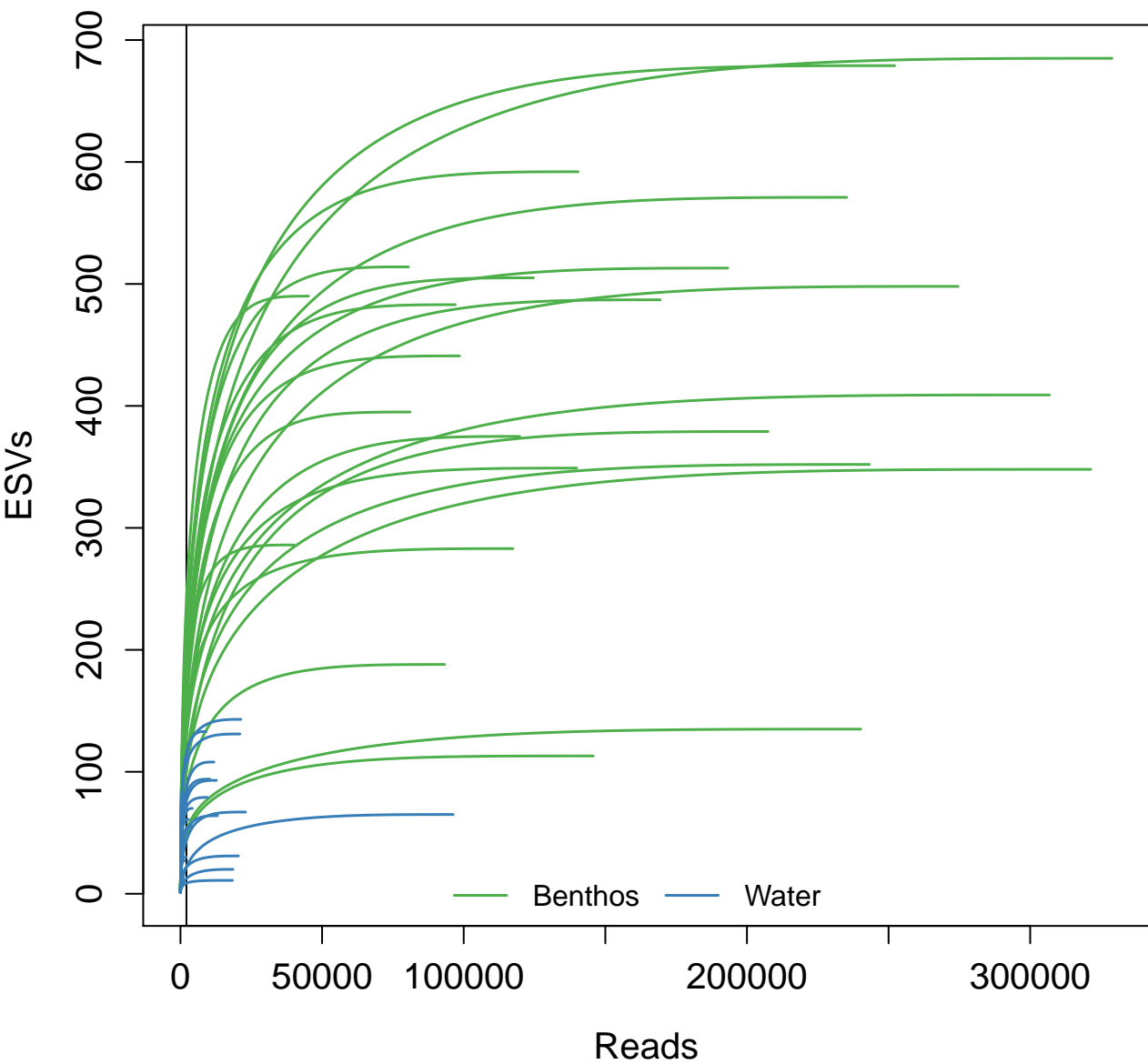

Supplement: S1 Fig — Benthos samples from each site are shown in green and water samples are shown in blue. The vertical line shows the number of reads that would be included after normalizing library size down to the 15th percentile (reads = 2,099). (PDF) [file pone.0225409.s005.pdf]

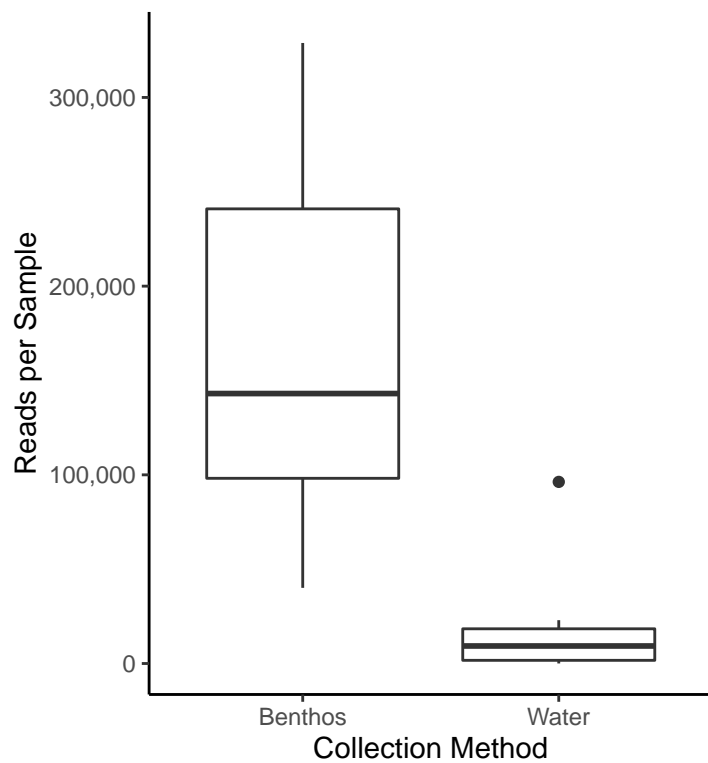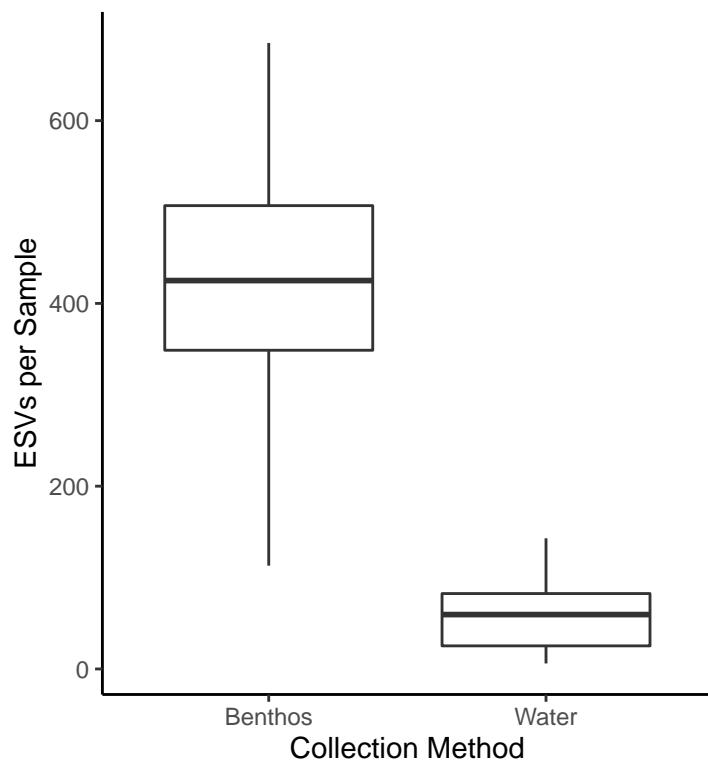

Supplement: S2 Fig — Results summarized before normalization. (PDF) [file pone.0225409.s006.pdf]

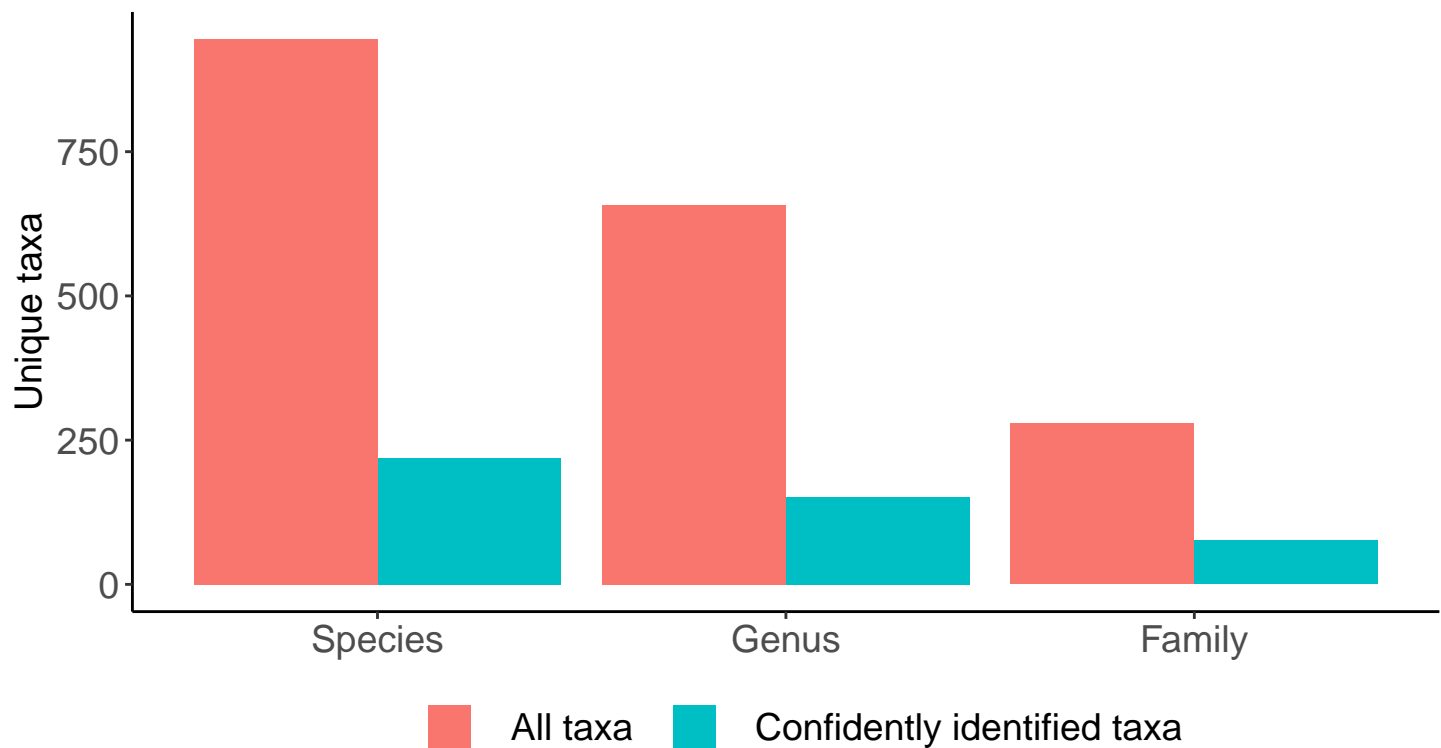

Supplement: S3 Fig — Results summarized before normalization. (PDF) [file pone.0225409.s007.pdf]

# Benthos

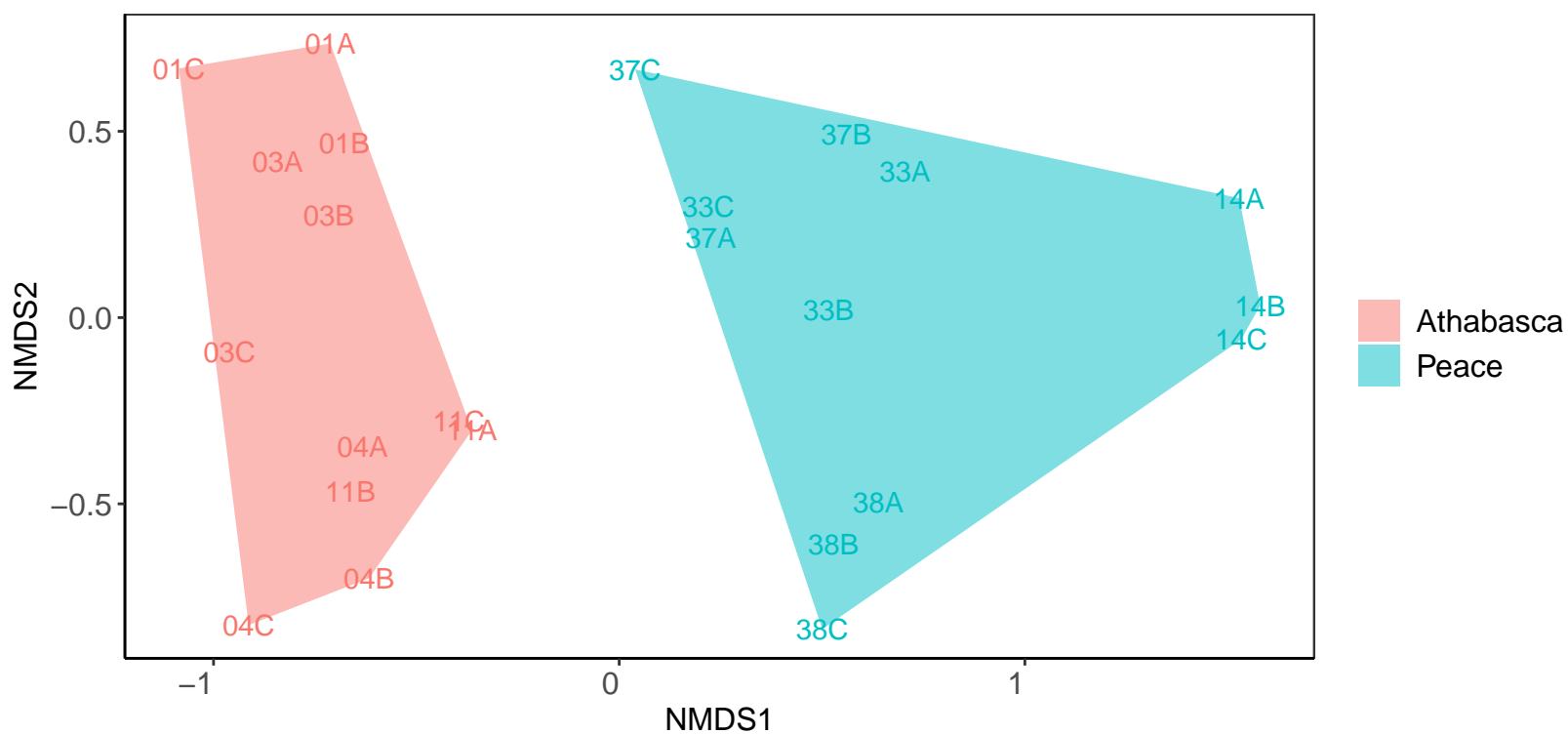

# Water

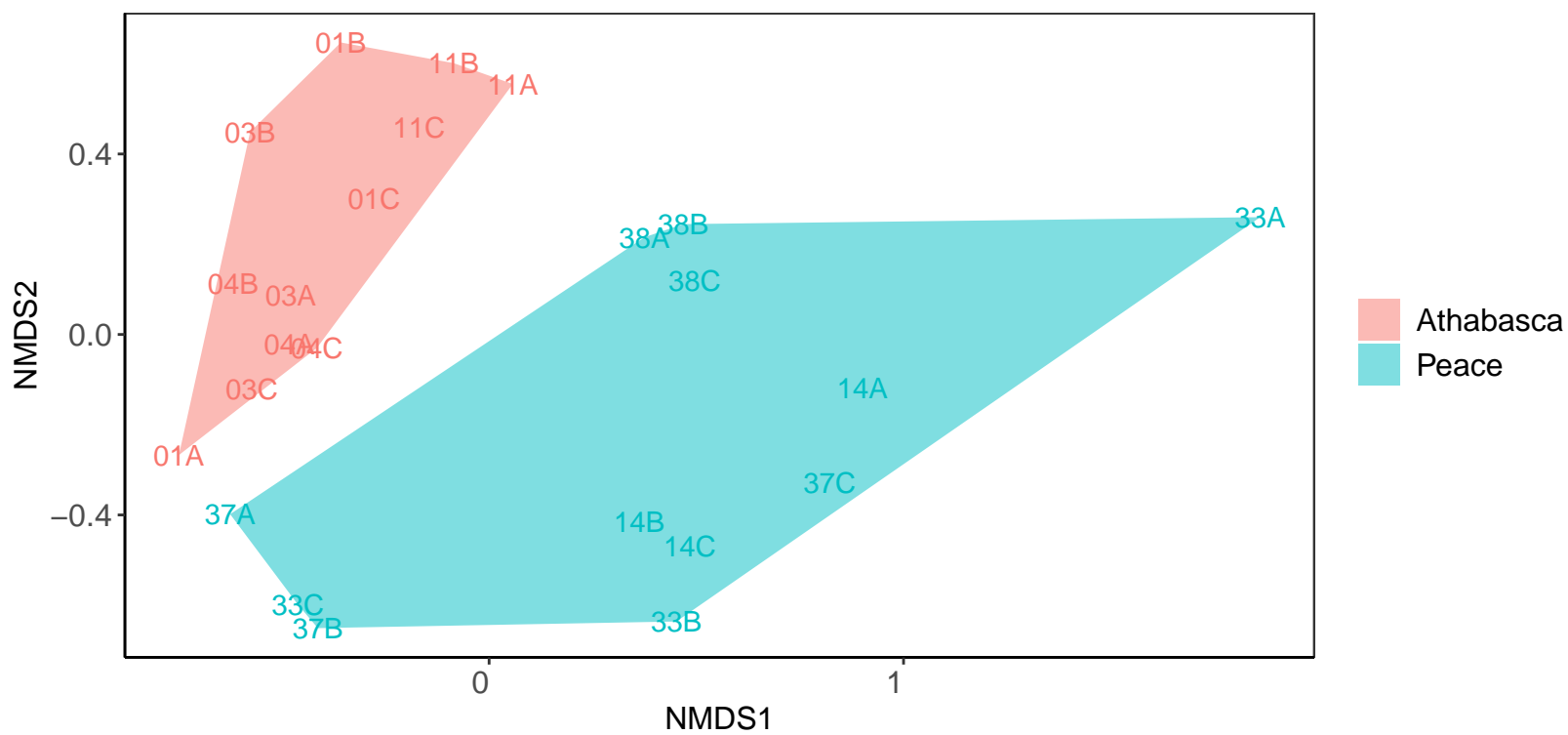

Supplement: S4 Fig — NMDS ordination distances well-represent observed Sorensen dissimilarities (Benthos stress = 0.08, R2 = 0.95; Water stress = 0.09, R2 = 0.95). PERMANOVA shows that river groupings are significant and explain 14–19% of the variation in beta diversity (Benthos R2 = 0.19, p-value = 0.001; Water R2 = 0.14, p-value = 0.001). Results based on normalized data. (PDF) [file pone.0225409.s008.pdf]
